# Supplementary material for: Wafer-sized multifunctional polyimine-based two-dimensional conjugated polymers with high mechanical stiffness
Source: Nat Commun. 2016 Nov 16;7:13461. doi: 10.1038/ncomms13461 (PMC5116084; doi:10.1038/ncomms13461)
Supplement: Supplementary Information — Supplementary Figures 1-9, Supplementary Tables 1 and 2, Supplementary Methods and Supplementary References [file ncomms13461-s1.pdf]

Supplementary Figures

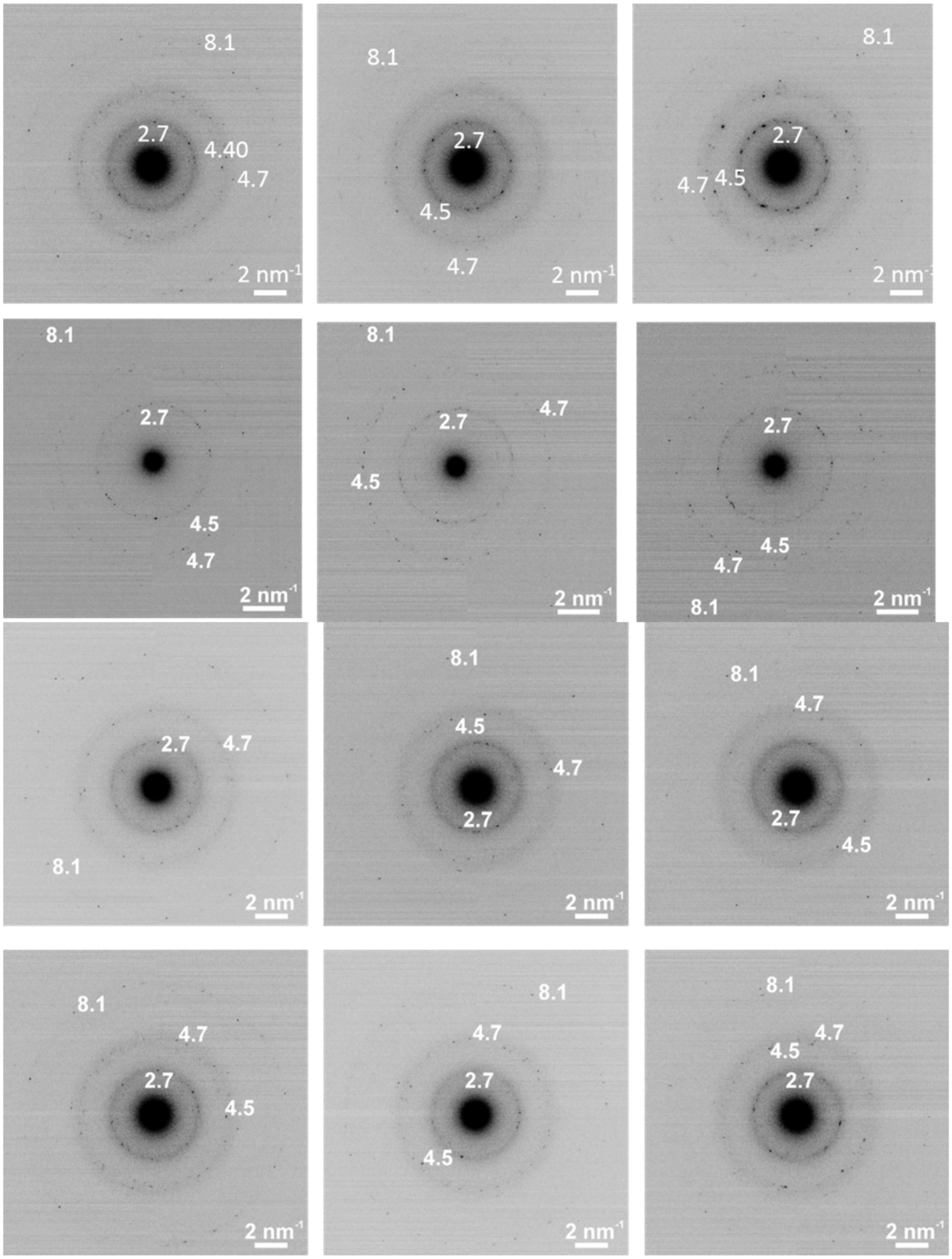

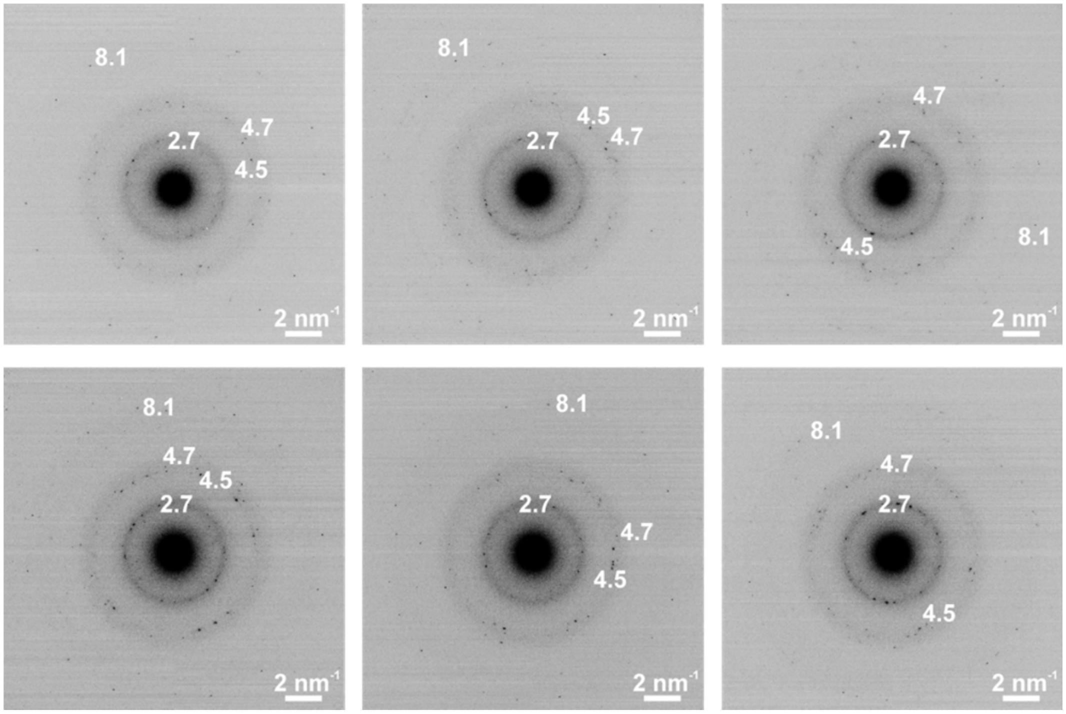

Supplementary Figure 1 │ Selected area electron diffraction patterns of monolayer 2DP (**4**) sandwiched by two layers of graphene (G), G/2DP/G.

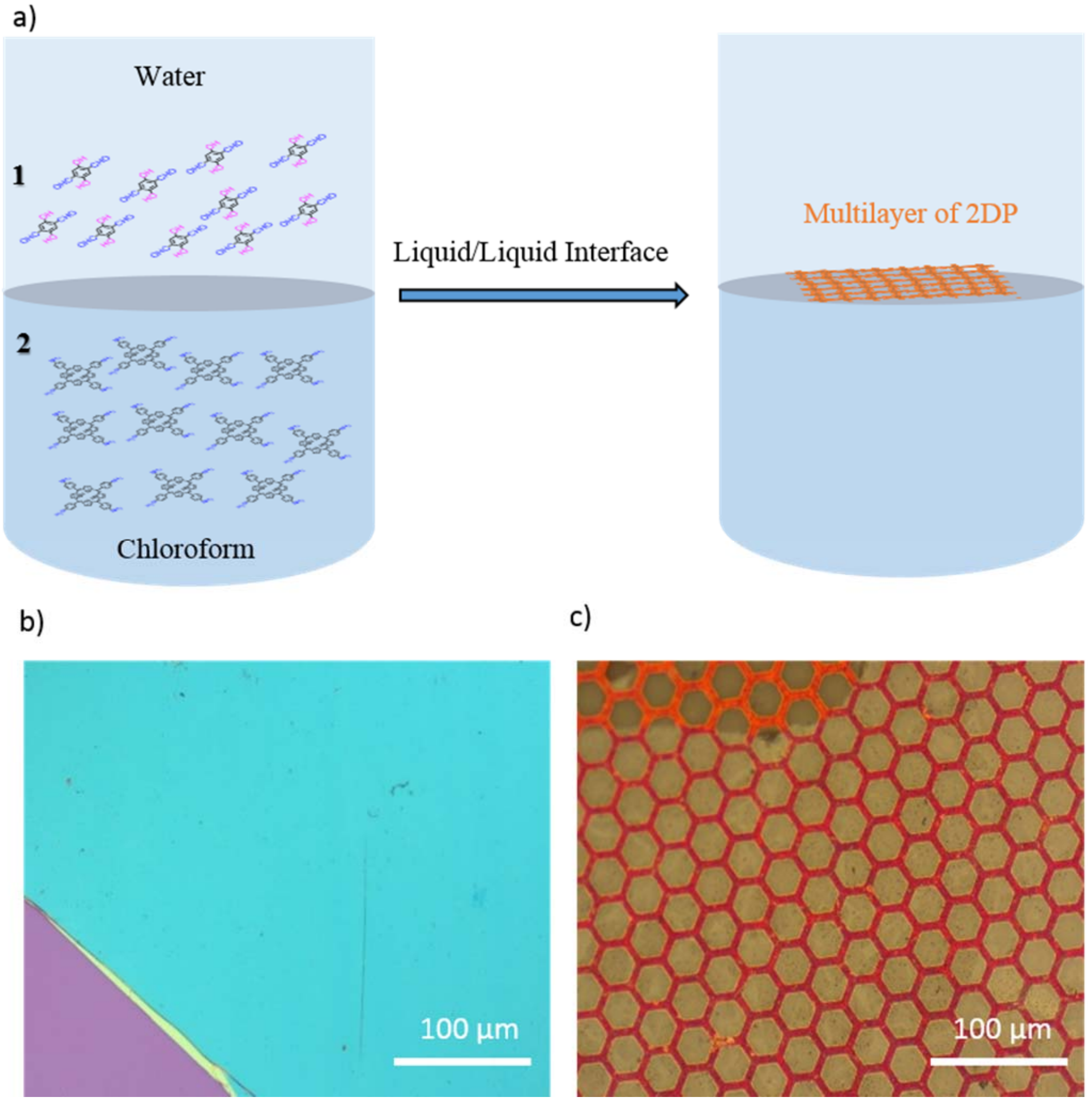

Supplementary Figure 2│ Schematic illustration for the synthesis and microscopic characterization of multilayer 2DP (**4**). a) Schematic illustration on the synthesis of multilayer of 2DP at water-chloroform interface. Optical images of the multilayer 2DP on b) 300 nm SiO_2/Si and c) copper grid, respectively.

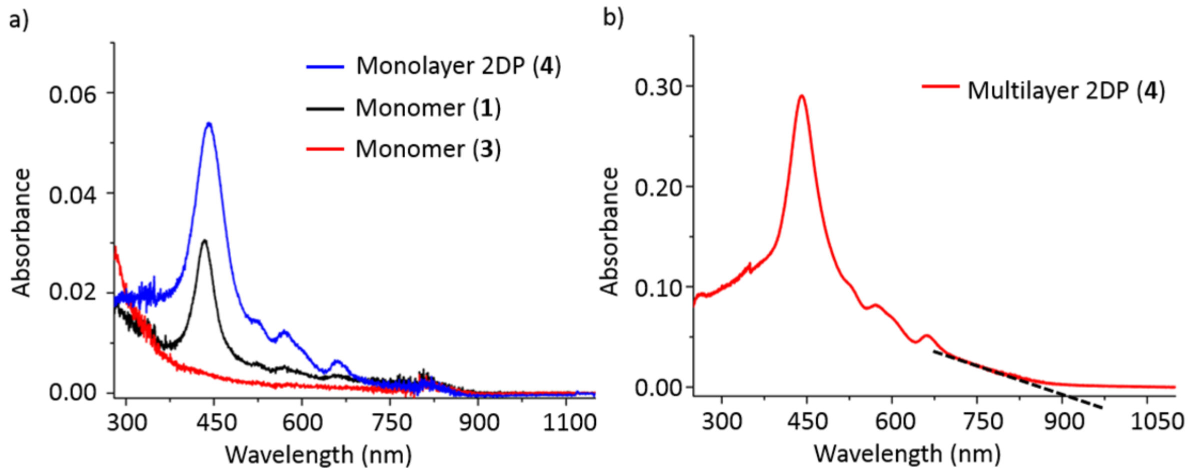

Supplementary Figure 3 | UV-vis spectroscopic characterization of monomers (**1**, **3**), monolayer and multilayer 2DP (**4**) on quartz. The peak at around 800 nm in a) was partially caused by variation due to detector change (at 800 nm). To eliminate the effect, multilayer 2DP (**4**) was explored for increasing the signal-to-noise ratio.

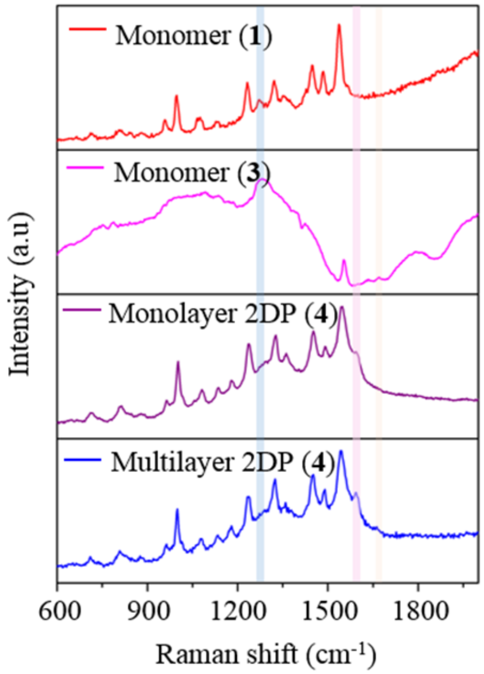

Supplementary Figure 4 | Raman spectra of monomers (**1**, **3**) and monolayer and multilayer of 2DPs (**4**) on 300 nm SiO_2/Si.

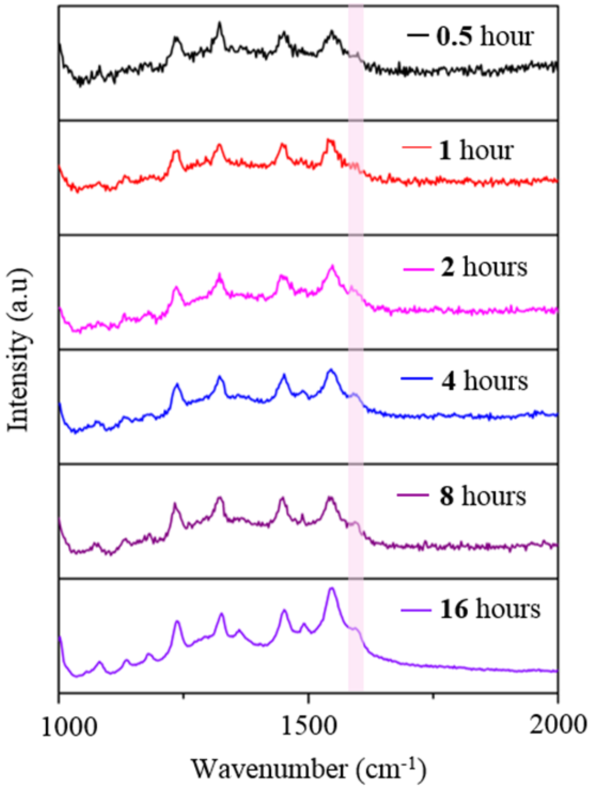

Supplementary Figure 5 | Raman spectra of monolayer 2DP (**4**) synthesized with different time intervals (30 minutes, 1 hour, 2 hours, 4 hours, 8 hours and 16 hours).

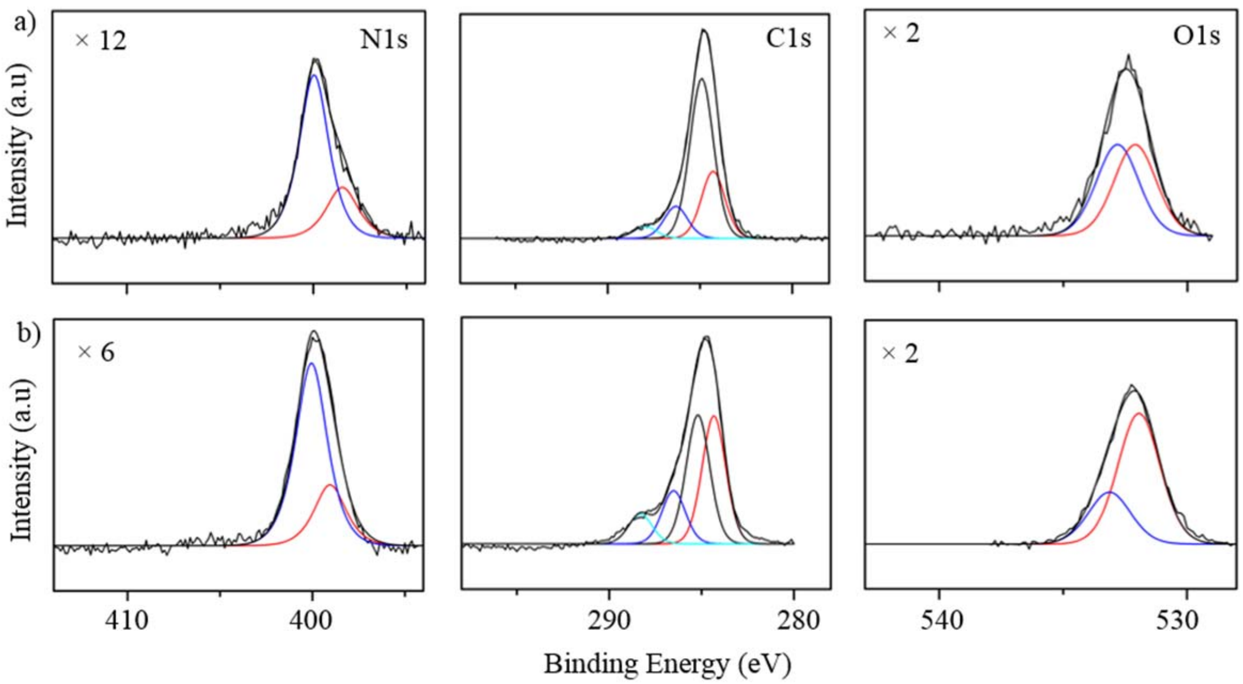

Supplementary Figure 6 │ XPS spectra of a) monolayer and b) multilayer of 2DPs (**4**) on Au/Si. The C1s signal consisted of four peaks with BEs at 284.2, 285.0, 286.3, and 287.0 eV, which are attributed to aromatic carbons ($C_{aromatic}$: porphyrin units, phenyl rings and C=N), air contaminations, C-N/C-O bond, and satellites of aromatic carbons, respectively. The O1s at a binding energy of ~ 533 eV and 532 eV are due to air contamination, and water and hydroxyl groups in the 2DPs, respectively. The spectra N1s of the monolayer and multilayer of 2DP were expanded by a factor of 12 and 6, respectively. The spectra of O1s were expanded by a factor of 2.

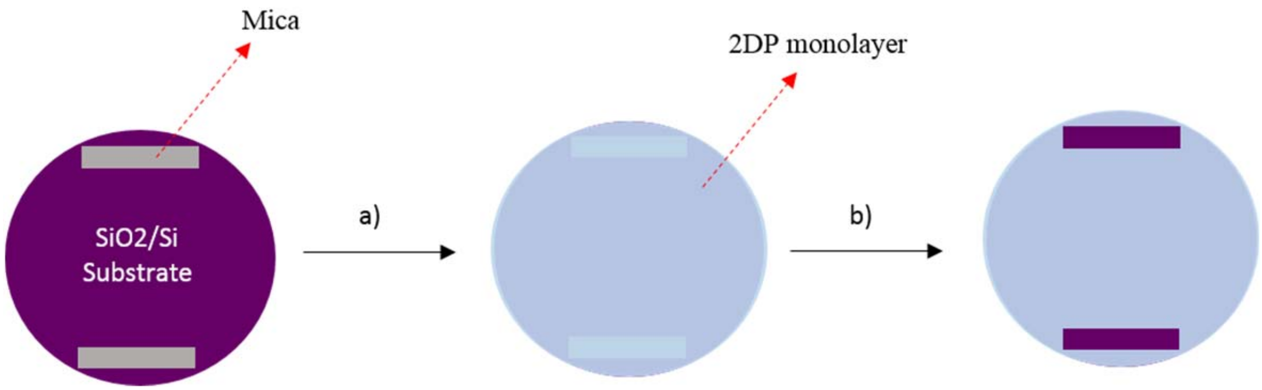

Supplementary Figure 7│Schematic illustration for the transfer of monolayer 2DP onto 4-inch silicon wafer for photograph in Figure 2i in the main text. a) Mica was placed on the edge of two lateral side of 4-inch Si wafer, followed by a horizontal transfer of the 2DP onto the sample surface, b) The placed mica was then removed, showing clear edges between the substrate and the 2DP.

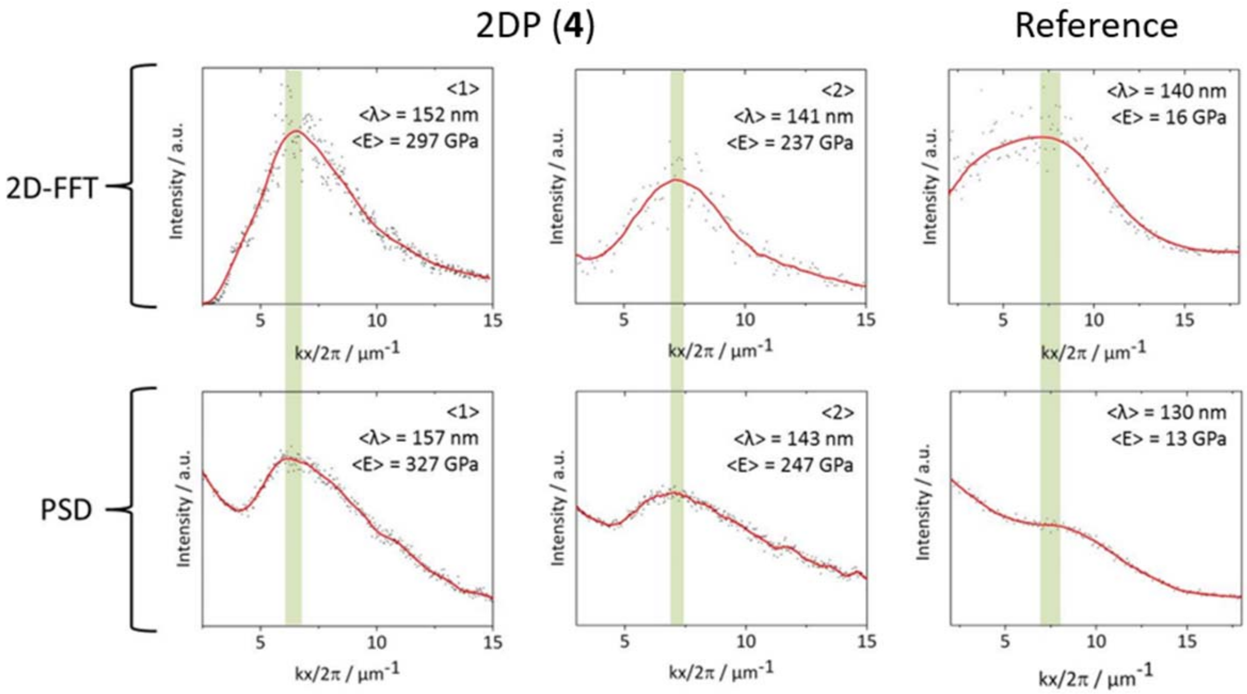

Supplementary Figure 8│ Wavelength estimation of 2DP (**4**) and bis(terpyridine)-metal complex nanosheet (reference) wrinkling by 2D Fourier-transformation (2D-FT) and Power spectral density (PSD) methods. Red lines correspond to integration perpendicular to the wrinkle direction. For the 2DP (**4**), a spectral analysis perpendicular to the wrinkles by 2D-FT and PSD exhibited a wavelength of 0.141 µm to 0.152 µm (top, left) and 0.143 µm to 0.157 µm (bottom, left), respectively. Both techniques are in close agreement for the wrinkle periodicity. While 2D-FT gives a Young's modulus E_{Young} of 237 – 297 GPa, PSD provides a value of 247 – 327 GPa. In average, the E_{Young} of the 2DP is 277 ± 37 GPa. For the bis(terpyridine)-metal complex nanosheet (E_{Young} = ~ 16 GPa from AFM nanoindentation reported by Schlüter et al)[1], a wavelength of 140 nm (top, right) and 130 nm (bottom, right) for 2D-FT and PSD, which gives a Young's modulus of ~ 16 GPa and 13 GPa, respectively.

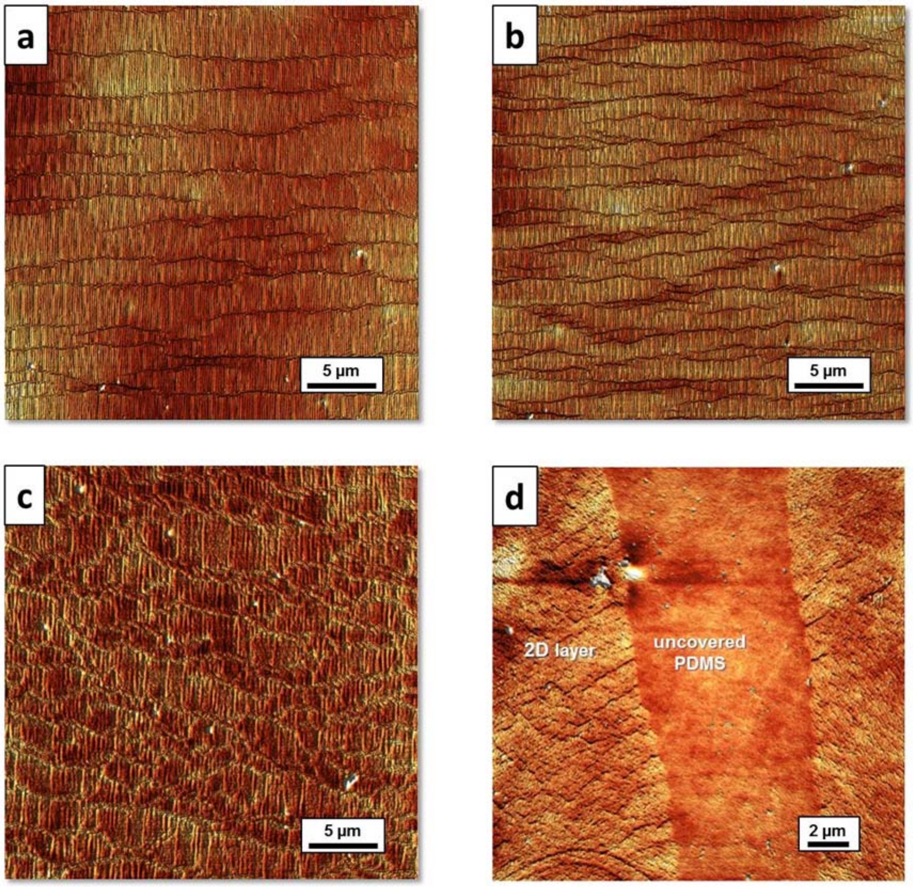

Supplementary Figure 9 | AFM z-senor images (a) 2DP layer, sample 1 (b) 2DP layer, sample 2 (c) reference sample and (d) unstrained 2DP layer at its interface to an uncovered region. All images have been made in 1024 x 1024 px high resolution to ensure statistical relevance.

Supplementary Tables

Supplementary Table 1: Comparison of the HER activity between Co-2DP (**5**) in the present work and molecular catalysts as well as doped graphene in basic solutions reported in the literature.

**Catalyst**	**Onset Potential (mV vs. RHE)**	**Tafel slope (mV dec^{-1})**	**Overpotential (mV)** @ 1mA cm^{-2}	Ref.
Co-2DP (**5**)	308	126	367 @ 1 mA	This work
Co-porphyrin embedded in graphene oxide sheets:ERGO@ CoTMPyP]$_7$	∼360	116	474	2
Diimine-dioxime cobalt catalyst	450	160	590	3
Dithiolene-nickel complex based 2D supramolecular polymer	360	-	∼ 370	4
P-doped graphene	650	159	∼ 575	
N-doped graphene	∼ 570	143	∼ 525	5
N,P doped graphene	∼ 540	145	∼ 480	
Cobalt Dithiolene Films	480	189	∼ 510	6
Nickel-sulfur complex based film and mesoporous MOF (NU-1000) hybrid structure	∼ 580	123	∼ 450	7
Ni-complexes	∼ 420	-	∼ 420	8

Supplementary Table 2 | Overview of SIEBIMM applications from literature and own work on the topic (green marked).

Sample	Layer thickness /nm	Periodicity /nm	In-Plane-Modulus /GPa	Reference
PS / Silica on PDMS (varying thicknesses)	150 – 250 nm	8 – 13 µm	3,4 GPa	9
Nanofibrillated cellulose on PEI (varying thicknesses)	35 – 75 nm	1 – 3 µm	1,5 GPa	10
Hectorite on PDMS	0,96 nm	149 nm	143 GPa	11
Graphene Oxide on PDMS	0,7 nm	89 nm<br>138 nm	110 GPa<br>420 GPa	12
Aluminium chlorohydrate on PDMS (while drying)	~ 5 nm	40 – 70 µm	10 – 190 MPa	13

Supplementary Methods

Mechanical characterization. To determine the stiffness of the 2DP (**4**) and bis(terpyridine)-metal complex nanosheet, we choose a simple and easy-to-handle metrology technique called Strain-Induced Elastic Buckling Instability for Mechanical Measurements (SIEBIMM), that is based on the buckling of thin films (wrinkling) on thick elastic supports[14,15]. For this purpose, a thin film needs to be situated on top of a thick elastomeric substrate in strong adhesive contact. Compressing this system induces wrinkling of the film. Analysis of the wrinkling frequency allows for the precise evaluation of the in-plane moduli of the thin layer. The wrinkle periodicity can be correlated to the mechanical mismatch of the top and supporting materials[16,17] as described in Supplementary Equation 1.

$$\lambda = 2\pi h_l \left[\frac{(1-\nu_s{}^2)E_l}{(1-\nu_l{}^2)E_s}\right]^{1/3} \qquad (1)$$

where h_f being the film's thickness, ν_s and ν_f are Poisson ratios as well as E_s and E_f are Young's Moduli of substrate and the film, respectively. Solving for E_f gives the in-plane modulus of the thin layer for a corresponding wavelength λ, as described in Supplementary Equation 2.

$$E_f = \frac{3E_s\,(1-\nu_l{}^2)}{(1-\nu_s{}^2)}\left(\frac{\lambda}{2\pi h_l}\right)^3 \qquad (2)$$

This buckling based metrology method has been investigated and applied to a variety of other thin films and 2D materials (see Supplementary Table 2). As can be seen, it is valid for a broad range of layer thicknesses as well as mechanical constants of both layer and substrate.

In the case investigated in this manuscript, we performed two sets of SIEBIMM experiments on identically prepared samples. After transfer of the 2D-layers onto the elastomer special care was taken to ensure strong adhesive contact, since weak adhesion will result in slippage and consequently an underestimation of Young's modulus. To avoid such a delamination, the transferred layer is dried cautiously over night at 50 °C, as it carries along a thin water layer from the LB-bath preparation. This hinders the adhesion of layer and elastomer, so the sample is not strained before any water has been evaporated. Subsequently it becomes clamped into a custom-made stretching-device and gets pulled longitudinally causing a laterally compression of both elastomer and layer. At a certain compression periodic wrinkle corrugations of the layer show up. For sample 1, the critical compression εc is at 8 %, for sample 2 we got 9 %. Subsequently the corrugations are imaged via AFM. For each set of experiments several 1024 x 1024 px high resolution AFM images of the wrinkled film in different states of compression were obtained, as shown in Supplementary Figure 9. Wrinkles and cracks perpendicular to the wrinkles can be clearly identified and areas which are not covered by the 2D-layer are wrinkle-free.

To statistically average our results, we performed two independent averaging methods, 2D-Fast Fourier Transformation (2D-FFT) and Power Spectral Density (PSD) analysis, which yield good agreement. 2D-FFT was performed by transferring the raw data into a 2D-FT image in Gwyddion 2.43 SPM analyzes

software. By choosing the relevant area, an angle correction of the data was performed simultaneously. The extracted cross-section then was load into Origin 2015 software and peak picking was performed. For PSD the integrated tool of Bruker NanoScope 1.6 software was used, which transfers and integrates the relevant area at the same time. Eventually the maximum peak is picked. Nevertheless, this method does not have angle correction of the raw data, wherefore larger values for the wrinkle periodicities are observable. We prevented this by only taking images into account that are already very close to a 90°-angle referring to the transfer direction. Supplementary Figure 10, shows a typical result for a representative sample. One can clearly identify a peak corresponding to the wrinkle wavelength.

An average over all samples resulted in wavelength of 146 nm (2D-FFT) and 150 nm (PSD). This corresponds to Young's moduli of 267 ± 30 GPa (2D-FFT) and 287 ± 40 GPa (PSD). Since generally 2D-FFT showed the periodicity more precisely due to its angle independency, but also clearly with a narrower peak, showed best agreement with literature in the reference experiments (see below) and is used in most studies in the literature, we report 267 ± 30 GPa in the manuscript.

Application of the method to a reference system. In an additional experiment we verified the data by wrinkling and measuring a thin 2DP film material that is very similar in the preparation, transfer of the film and adhesion treatment of the film and which has been previously characterized by an independent method[1] (AFM force spectroscopy). Here, SIEBIMM analysis results a Young's Modulus of 16 GPa (2D-FFT) and 13 GPa (PSD). Both values are in very good agreement with the primal AFM Nano-indentation result of 16 GPa, altogether proving the reliability of our investigation.

Microscopic characterization. Optical images were acquired in differential interference mode with AxioScope A1, Zeiss. Atomic force microscopy (AFM) was performed on a customized Ntegra Aura/Spectra from NT-MDT (Moscow, Russia) with a SMENA head in contact mode. The probes have a typical curvature radius of 6 nm, a resonant frequency of 47−150 kHz, and a force constant of 0.35−6.10 N/m. Scanning electron microscopy (SEM) was conducted on Helios NanoLab 660 operated at an acceleration voltage of 10kV with an retractable STEM detector. In order to investigate the structure of the 2DP (**4**), electron diffraction experiments were carried out using an aberration-corrected FEI Titan 80-300 transmission electron microscope operated at an acceleration voltage of 80 kV.

Spectroscopic characterization. UV-vis spectroscopy was performed on NR 5000 (Aglient technologies, Germany). Spectra of the 2DPs (**4**) on quartz glass were recorded in transmission mode. Raman spectra were acquired on confocal Raman microscope (NT-MDT). The Raman spectrum was excited by a 532 nm (2.33 eV) laser, and the spot size of the laser beam was about 0.5μm. XPS measurements were performed

with AXIS Ultra DLD system from Kratos with Al Kα radiation as X-ray source of (1486.6 eV) twin anode run at 10mA and 40 kV.

Mobility calculation. Field effect mobility μ was calculated based on the linear regime (μ_{lin}) of transistor transfer plots (ISD vs. VG), by the equation[18].

$$I_{SD}=\mu_{lin}\frac{W}{L}\,Ci\,(V_G-V_T)\,V_{SD} \qquad (3)$$

Where I_{SD} is the source-drain current, W and L are the width and length of the channel, C_i is the gate dielectric capacitance, V_G is the gate voltage, V_T is the threshold voltage, and V_{SD} is the source-drain voltage.

Theory calculation. All calculations are performed using the SCC-DFTB (self-consistent charge density functional based tight-binding) method[19-22]. The SCC-DFTB method is based on the Taylor expansion of the DFT Kohn-Sham energy in terms of the charge density fluctuations. The Hamiltonian is calculated using a minimal basis set of pseudoatomic orbitals with a two-center approximation. The SCC-DFTB method has been used as implemented in the DFTB+ program[19-21] and it has been successfully applied for calculating different electronic or optical properties of large molecules, clusters or periodic systems, which gave similar results to those obtained by *ab initio* DFT method at lower computational effort[23]. When the SCC-DFTB method was employed for investigating the electronic properties of the 2DP (**4**) structures, we have optimized the atomic positions for all the structures and after that the density of states (DOS) calculations have been performed.

Supplementary References:

1. Zheng, Z. *et al*. Square-micrometer-sized, free-standing organometallic sheets and their square-centimeter-sized multilayers on solid substrates. *Macromol. Rapid Commun*. ***34***, 1670-1680 (2013).

2. Huang, D. et al. Fabrication of cobalt porphyrin. Eectrochemically reduced graphene oxide hybrid films for electrocatalytic hydrogen evolution in aqueous solution. *Langmuir* **30**, 6990-6998 (2014).

3. Andreiadis, E. S. et al. Molecular engineering of a cobalt-based electrocatalytic nanomaterial for H_2 evolution under fully aqueous conditions. *Nat. Chem*. **5**, 48-53 (2013).

4. Dong, R. *et al.* Large-area, free-standing, two-dimensional supramolecular polymer single-layer sheets for highly efficient electrocatalytic hydrogen evolution. *Angew. Chem.* **127**, 12226-12231 (2015)

5. Zheng, Y. *et al.* Toward design of synergistically active carbon-based catalysts for electrocatalytic hydrogen evolution. *ACS Nano* **8**, 5290-5296 (2014).

6.. Clough, A. J., Yoo, J. W., Mecklenburg, M. H. & Marinescu, S. C. Two-dimensional metal–organic surfaces for efficient hydrogen evolution from water. *J. Am. Chem. Soc.* **137**, 118-121 (2015).

7. Hod, I. *et al.* A porous proton-relaying metal-organic framework material that accelerates electrochemical hydrogen evolution. *Nat. Commun.* **6**, 8304 (2015).

8. Das, A., Han, Z., Brennessel, W. W., Holland, P. L. & Eisenberg, R. Nickel complexes for robust light driven and electrocatalytic hydrogen production from water. *ACS Catal.* **5**, 1397-1406 (2015).

9. Stafford, C.M et.al. A buckling-based metrology for measuring the elastic moduli of polymeric thin films. *Nat. Mater*. **3**, 545-550 (2004).

10. Wägberg, L. *et al*. Determination of Young's Modulus for Nanofibrillated Cellulose Multilayer Thin Films Using Buckling Mechanics, *Biomacromolecules* **12**, 961-969 (2011).

11. Breu, J. *et al*. In-Plane Modulus of Singular 2:1 Clay Lamellae Applying a Simple Wrinkling Technique, *ACS Appl. Mater. Interfaces* **5**, 5851-5855 (2013).

12. Kunz, D. A. *et al.* Space-Resolved In-Plane Moduli of Graphene Oxide and Chemically Derived Graphene Applying a Simple Wrinkling Procedure. *Adv. Mater.* **25,** 1337–1341 (2013).

13. Cabral, J.T. et al. Wrinkling Measurement of the Mechanical Properties of Drying Salt Thin Films, *Langmuir* **32**, 2199-2207 (2016).

14. Stafford, C. M. et al. A buckling-based metrology for measuring the elastic moduli of polymeric thin films. *Nature Materials* **3**, 545-550 (2004).

14. Reyes-Martinez, M. A., Ramasubramaniam, A., Briseno, A. L. & Crosby, A. J. The intrinsic mechanical properties of rubrene single crystals. *Adv. Mater*. **24**, 5548-5552 (2012).

16. Bowden, N., Huck, W. T. S., Paul, K. E. & Whitesides G. M. The controlled formation of ordered, sinusoidal structures by plasma xxidation of an elastomeric polymer. *Appl. Phys. Lett.* **75**, 2557-2559 (1999).

17. Glatz, B. A. et al. Hierarchical line-defect patterns in wrinkled surfaces. *Soft Matter* **11**, 3332-3339 (2015).

18. Sirringhaus, H. 25th Anniversary Article: Organic Field-Effect Transistors: The path beyond amorphous silicon. *Adv. Mater.* **26,** 1319-1335 (2014).

19. Porezag, D., Frauenheim, T., Köhler, T., Seifert, G. & Kaschner, R. Construction of tight-binding-like potentials on the basis of density-functional theory: Application to carbon. *Phys. Rev. B* **51**, 12947-12957 (1995).

20. Elstner, M. et al. Self-consistent-charge density-functional tight-binding method for simulations of complex materials properties. *Phys. Rev. B* **58**, 7260-7268 (1998).

21. Seifert, G. Tight-Binding Density Functional Theory: An approximate Kohn−Sham DFT scheme. *J. Phys. Chem. A* **111**, 5609-5613 (2007).

22. Liang, R., Swanson, J. M. J. & Voth, G. A. Benchmark study of the SCC-DFTB approach for a Biomolecular Proton Channel. *J. Chem. Theory Comput*. **10**, 451-462 (2014).

23. Elstner, M.; Frauenheim, T.; McKelvey, J.; Seifert, G. Density functional tight binding: Contributions from the American Chemical Society Symposium. *J. Phys. Chem. A* **111**, 5607-5608 (2007).
